# Supplementary material for: Molecular parallelisms between pigmentation in the avian iris and the integument of ectothermic vertebrates
Source: PLoS Genet. 2021 Feb 23;17(2):e1009404. doi: 10.1371/journal.pgen.1009404 (PMC7935293; doi:10.1371/journal.pgen.1009404)

S1 Fig. Alignment of multiple bird genomes in the candidate nonsense mutation region. The candidate mutation is highlighted in orange.

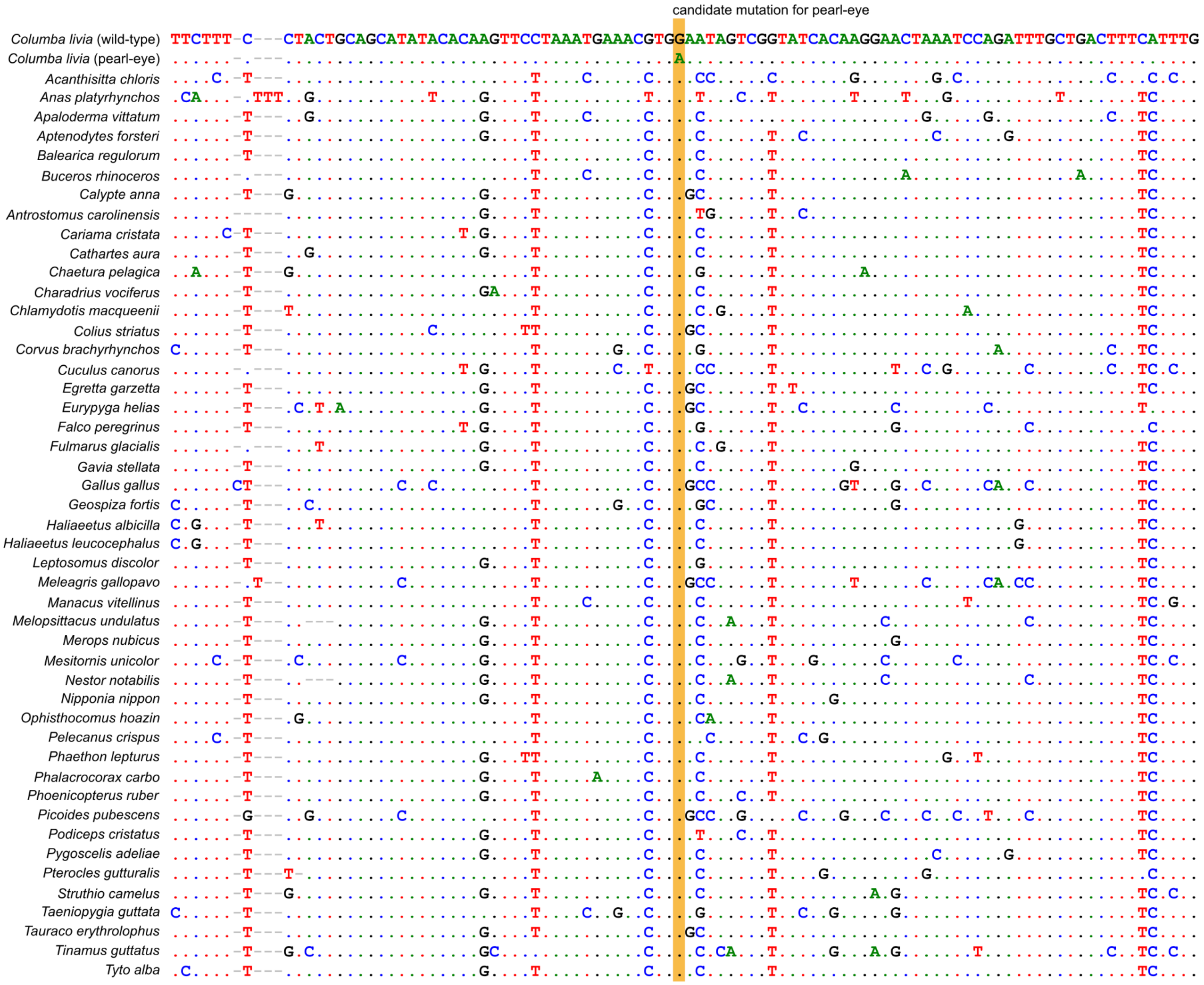

Supplement: S1 Fig — The candidate mutation is highlighted in orange. (PDF) [file pgen.1009404.s001.pdf]
