# Supplementary material for: Molecular parallelisms between pigmentation in the avian iris and the integument of ectothermic vertebrates
Source: PLoS Genet. 2021 Feb 23;17(2):e1009404. doi: 10.1371/journal.pgen.1009404 (PMC7935293; doi:10.1371/journal.pgen.1009404)

S2 Fig. Exon structure of *SLC2A11B* from one wild-type and one pearl-eye homozygous pigeons that were used for transcriptome assembly. **(A)** Alignment of assembled transcripts of *SLC2A11B* to zebra finch and chicken transcripts from the *Ensembl* database (retrieved on November 2020). The putative canonical transcript of *SLC2A11B* in pigeons, and its respective predicted open reading frame, is shown on top. Of all the transcripts that were assembled, we only considered those that represented more than 10% of the overall transcripts mapping to the candidate region. Colors indicate the base content of each sequence: adenine – green; guanine – black; cytosine – blue; thymine – red. **(B)** Sashimi plots based on reads mapping to the genomic interval containing *SLC2A11B* for the two pigeons. Compared to the wild-type, the pearl-eye individual has a relatively decreased amount of reads in the 5' end of the gene, different proportions of reads along the transcript and relatively increased amount of reads in intronic regions.

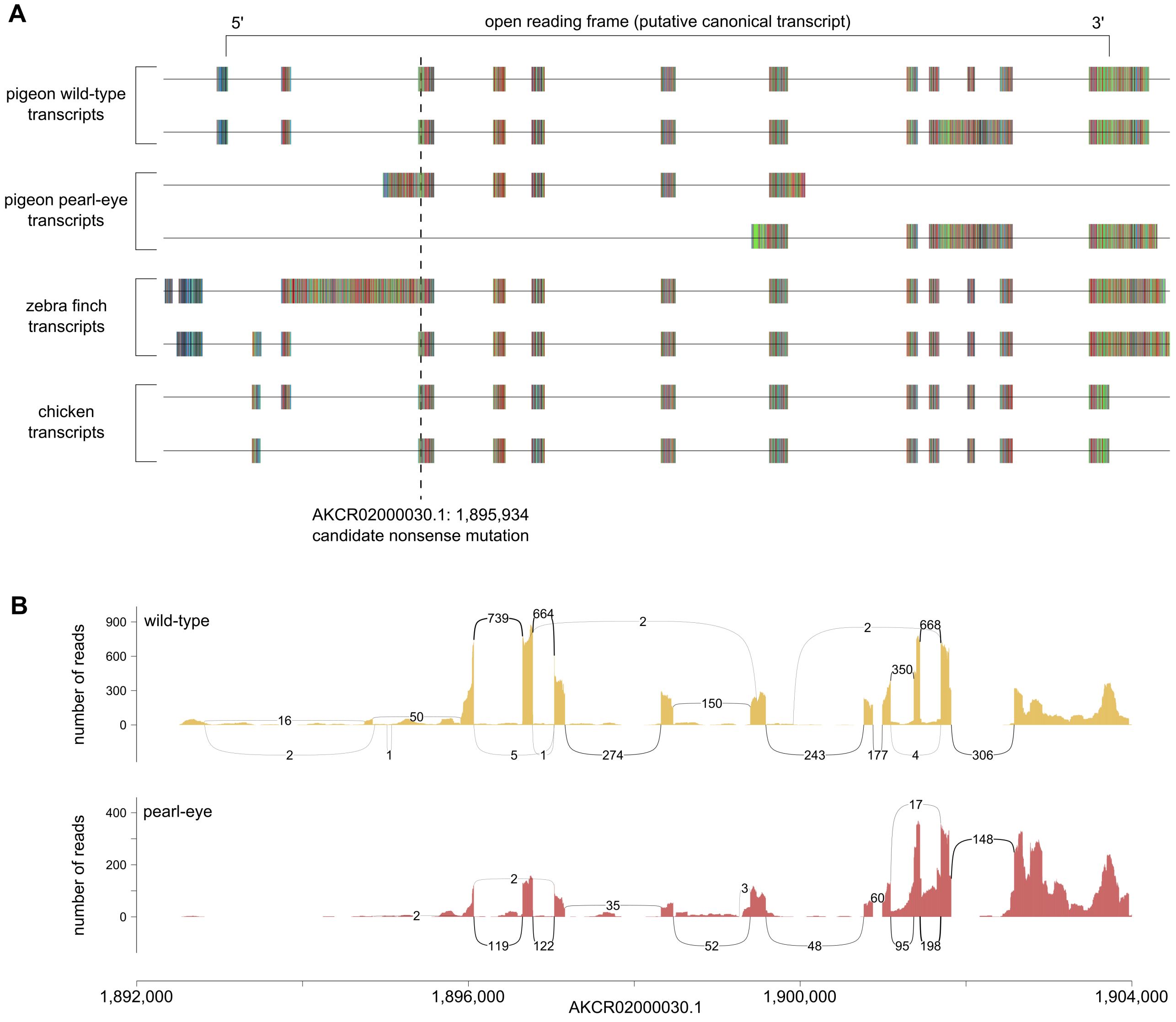

Supplement: S2 Fig — (A) Alignment of assembled transcripts of SLC2A11B to zebra finch and chicken transcripts from the Ensembl database (retrieved on November 2020). The putative canonical transcript of SLC2A11B in pigeons, and its respective predicted open reading frame, is shown on top. Of all the transcripts that were assembled, we only considered those that represented more than 10% of the overall transcripts mapping to the candidate region. Colors indicate the base content of each sequence: adenine–green; guanine–black; cytosine–blue; thymine–red. (B) Sashimi plots based on reads mapping to the genomic interval containing SLC2A11B for the two pigeons. Compared to the wild-type, the pearl-eye individual has a relatively decreased amount of reads in the 5’ end of the gene, different proportions of reads along the transcript and relatively increased amount of reads in intronic regions. (PDF) [file pgen.1009404.s002.pdf]
