# Supplementary material for: Molecular parallelisms between pigmentation in the avian iris and the integument of ectothermic vertebrates
Source: PLoS Genet. 2021 Feb 23;17(2):e1009404. doi: 10.1371/journal.pgen.1009404 (PMC7935293; doi:10.1371/journal.pgen.1009404)

S3 Fig. Gene expression profiles between pearl-eye (red) and wild-type (yellow) pigeons for candidate genes for skin color development in ectothermic vertebrates. **(A)** Relative expression levels (TPM, transcripts per million) of genes in the pterin synthesis pathway. **(B)** Similar to panel (A) for a group of genes previously implicated in xanthophore differentiation in ectothermic vertebrates.

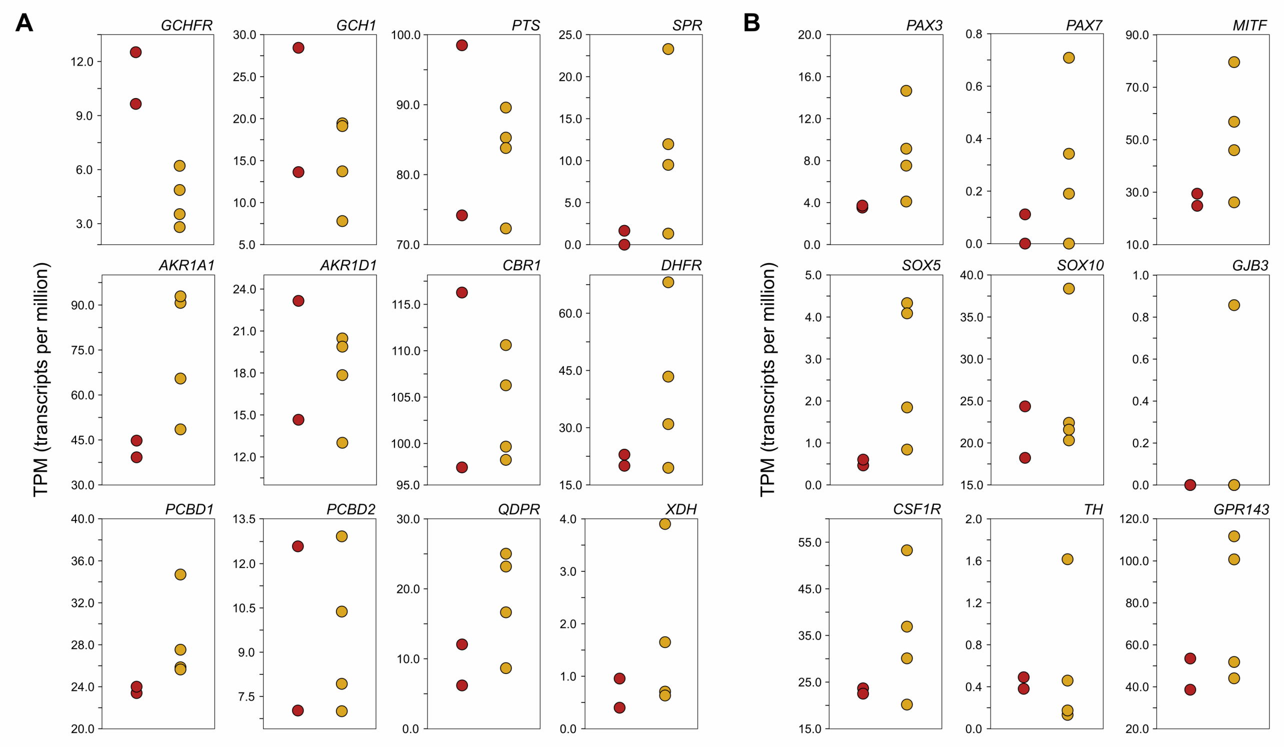

Supplement: S3 Fig — Gene expression profiles between pearl-eye (red) and wild-type (yellow) pigeons for candidate genes for skin color development in ectothermic vertebrates. (A) Relative expression levels (TPM, transcripts per million) of genes in the pterin synthesis pathway. (B) Similar to panel (A) for a group of genes previously implicated in xanthophore differentiation in ectothermic vertebrates. (PDF) [file pgen.1009404.s003.pdf]
