# Supplementary material for: Molecular parallelisms between pigmentation in the avian iris and the integument of ectothermic vertebrates
Source: PLoS Genet. 2021 Feb 23;17(2):e1009404. doi: 10.1371/journal.pgen.1009404 (PMC7935293; doi:10.1371/journal.pgen.1009404)
Supplement: S1 Table — (PDF) [file pgen.1009404.s004.pdf]

S1 Table. Sequencing summary statistics of samples used for whole-genome analyses.

Sample	Phenotype	Number of reads	Percentage of reads mapping (% properly paired)	Depth of coverage	Source of data
African owl	wild-type	173,269,178	98.60% (82.84%)	11.74	Shapiro et al. 2013
Barb	pearl-eye	185,474,172	98.86% (86.04%)	12.78	Shapiro et al. 2013
Berlin Longface tumbler	pearl-eye	169,052,395	98.95% (86.10%)	11.86	Shapiro et al. 2013
Birmingham roller	pearl-eye	154,312,471	98.32% (82.08%)	10.41	Shapiro et al. 2013
Capuchine	pearl-eye	130,038,779	98.78% (92.32%)	13.26	Shapiro et al. 2013
Carneau	pearl-eye	187,229,032	97.93% (81.68%)	12.19	Shapiro et al. 2013
Chinese owl	wild-type	143,024,193	98.47% (81.50%)	9.40	Shapiro et al. 2013
Cumulet	pearl-eye	161,028,200	99.02% (86.18%)	11.34	Shapiro et al. 2013
Dewlap	wild-type	190,474,160	99.12% (83.54%)	13.02	Shapiro et al. 2013
English carrier	wild-type	169,828,550	99.17% (83.85%)	12.07	Shapiro et al. 2013
English pouter	wild-type	181,082,994	98.21% (82.83%)	12.27	Shapiro et al. 2013
English trumpeter	wild-type	220,249,265	98.61% (83.52%)	14.75	Shapiro et al. 2013
English tumbler	pearl-eye	128,545,752	98.57% (83.83%)	8.84	Shapiro et al. 2013
Frillback	wild-type	201,071,108	99.21% (83.97%)	14.24	Shapiro et al. 2013
Jacobin	pearl-eye	142,153,711	98.80% (84.19%)	9.96	Shapiro et al. 2013
King	wild-type	188,217,742	98.81% (83.42%)	12.75	Shapiro et al. 2013
Marchenero	wild-type	215,517,315	98.39% (89.07%)	15.71	Shapiro et al. 2013
Oriental frill	wild-type	134,181,191	98.69% (81.77%)	8.10	Shapiro et al. 2013
Oriental roller	pearl-eye	139,191,535	98.25% (82.94%)	9.43	Shapiro et al. 2013
Parlor roller	pearl-eye	126,760,365	99.02% (82.52%)	8.68	Shapiro et al. 2013
Saxon pouer	wild-type	224,405,779	99.22% (84.47%)	15.92	Shapiro et al. 2013
Swift	wild-type	182,394,464	99.14% (81.83%)	12.23	Shapiro et al. 2013
pigeonGOLDEN01	wild-type	53,335,247	99.26% (96.85%)	6.15	this study
pigeonGOLDEN02	wild-type	82,803,568	99.31% (97.33%)	9.68	this study
pigeonGOLDEN03	wild-type	88,804,014	99.24% (97.04%)	10.3	this study
pigeonGOLDEN04	wild-type	215,598,145	99.56% (97.34%)	25.57	this study
pigeonGOLDEN05	wild-type	54,557,343	99.57% (97.09%)	6.48	this study
pigeonGOLDEN06	wild-type	103,423,810	99.55% (97.60%)	12.33	this study
pigeonGOLDEN07	wild-type	53,278,237	99.60% (97.71%)	6.34	this study
pigeonGOLDEN08	wild-type	51,686,262	99.26% (96.76%)	5.94	this study
pigeonGOLDEN09	wild-type	296,020,053	99.55% (97.54%)	35.37	this study
pigeonGOLDEN10	wild-type	45,105,900	99.46% (97.01%)	5.18	this study
pigeonGOLDEN11	wild-type	88,676,787	99.50% (97.54%)	10.35	this study
pigeonGOLDEN12	wild-type	49,553,471	99.48% (97.61%)	5.94	this study
pigeonGOLDEN13	wild-type	56,101,301	99.18% (96.76%)	6.56	this study
pigeonGOLDEN14	wild-type	49,013,227	98.84% (95.71%)	5.56	this study
pigeonWHITE01	pearl-eye	77,715,988	99.47% (97.52%)	9.09	this study
pigeonWHITE02	pearl-eye	53,381,471	99.58% (97.33%)	6.24	this study
pigeonWHITE03	pearl-eye	145,486,326	99.55% (97.38%)	17.34	this study
pigeonWHITE04	pearl-eye	122,687,100	99.23% (96.69%)	13.69	this study
pigeonWHITE05	pearl-eye	64,037,689	99.25% (97.37%)	7.46	this study
pigeonWHITE06	pearl-eye	82,557,408	99.26% (97.56%)	9.6	this study
pigeonWHITE07	pearl-eye	83,134,197	99.26% (97.79%)	9.73	this study
pigeonWHITE08	pearl-eye	82,285,232	99.37% (95.78%)	9.28	this study
pigeonWHITE09	pearl-eye	82,846,357	99.35% (97.17%)	9.43	this study
pigeonWHITE10	pearl-eye	296,258,378	99.31% (96.81%)	34.68	this study
pigeonWHITE11	pearl-eye	60,046,396	99.45% (97.41%)	7.2	this study
pigeonWHITE12	pearl-eye	95,854,728	99.46% (97.29%)	11.56	this study
pigeonWHITE13	pearl-eye	109,943,644	99.52% (97.66%)	13.3	this study
pigeonWHITE14	pearl-eye	57,792,666	99.05% (96.09%)	6.77	this study
pigeonWHITE15	pearl-eye	73,667,184	99.25% (96.87%)	8.71	this study
