# Supplementary material for: Molecular parallelisms between pigmentation in the avian iris and the integument of ectothermic vertebrates
Source: PLoS Genet. 2021 Feb 23;17(2):e1009404. doi: 10.1371/journal.pgen.1009404 (PMC7935293; doi:10.1371/journal.pgen.1009404)
Supplement: S2 Table — Genotypes were determined through Sanger sequencing. (PDF) [file pgen.1009404.s005.pdf]

S2 Table. Summary statistics of samples used for RNA sequencing and allele counts at the candidate causal mutation (AKCR02000030.1:1,895,934bp). Genotypes were determined through Sanger sequencing.

Sample	Phenotype	Genotype	Number of reads	Counts for allelic imbalance
pigeonWHITE1	pearl-eye	A/A	39,343,316	-
pigeonWHITE2	pearl-eye	A/A	80,639,482	-
pigeonYELLOW1	wild-type	G/G	67,159,062	-
pigeonYELLOW2	wild-type	G/A	138,052,048	G=53 / A=2
pigeonYELLOW3	wild-type	G/A	98,333,946	G=36 / A=10
pigeonYELLOW4	wild-type	G/A	135,116,756	G=63 / A=16

NOTE: G – wild-type allele; A – pearl-eye allele
