# Supplementary material for: Molecular parallelisms between pigmentation in the avian iris and the integument of ectothermic vertebrates
Source: PLoS Genet. 2021 Feb 23;17(2):e1009404. doi: 10.1371/journal.pgen.1009404 (PMC7935293; doi:10.1371/journal.pgen.1009404)
Supplement: S3 Table — Negative fold-change values indicate down-regulation in pearl-eye iris. Linkage group information follows the genetic map of [18]. Genes located in linkage group 20 (the same as the putative causal locus) are marked in bold. (PDF) [file pgen.1009404.s006.pdf]

S3 Table. List of differentially expressed genes between the iris of wild-type and pearl-eye pigeons. Negative fold-change values indicate down-regulation in pearl-eye iris. Linkage group information follows the genetic map of [18]. Genes located in linkage group 20 (the same as the putative causal locus) are marked in **bold**.

Transcript	Linkage group	Gene	Product	Log fold-change	P-value	FDR-corrected P
lcl|AKCR02000001.1_mrna_400	2	*FAM110B*	family with sequence similarity 110, member B, transcript variant X1	-7.44	4.71E-14	2.14E-10
lcl|AKCR02000001.1_mrna_502	2	*MIB1*	mindbomb E3 ubiquitin protein ligase 1, transcript variant X3	-6.49	1.14E-04	2.87E-02
lcl|AKCR02000001.1_mrna_740	2	*ZNF622*	zinc finger protein 622, transcript variant X1	-8.28	5.60E-07	4.77E-04
lcl|AKCR02000004.1_mrna_2854	2	*CAPN7*	calpain 7, transcript variant X1	-6.60	1.84E-05	8.35E-03
lcl|AKCR02000006.1_mrna_3665	4	*ACOX3*	acyl-CoA oxidase 3, pristanoyl, transcript variant X1	-5.79	5.09E-05	1.65E-02
lcl|AKCR02000006.1_mrna_3997	4	*RWDD4*	RWD domain containing 4, transcript variant X1	-2.37	1.01E-05	5.40E-03
lcl|AKCR02000008.1_mrna_4434	1	*PACSIN2*	protein kinase C and casein kinase substrate in neurons 2, transcript variant X2	-4.46	4.36E-05	1.61E-02
lcl|AKCR02000011.1_ncrna_5318	5	LOC102090964	uncharacterized LOC102090964, transcript variant X2	-7.34	1.76E-04	4.00E-02
lcl|AKCR02000011.1_mrna_5348	5	*AP2A2*	adaptor-related protein complex 2, alpha 2 subunit, transcript variant X1	-3.17	7.06E-05	2.15E-02
lcl|AKCR02000011.1_mrna_5471	5	*USP47*	ubiquitin specific peptidase 47, transcript variant X1	-7.22	1.89E-06	1.33E-03
lcl|AKCR02000013.1_mrna_5823	4	*SPP1*	secreted phosphoprotein 1	-2.73	7.39E-07	5.92E-04
lcl|AKCR02000014.1_mrna_5976	6	*CLASP1*	cytoplasmic linker associated protein 1, transcript variant X6	-4.22	3.71E-05	1.40E-02
lcl|AKCR02000014.1_mrna_6035	6	*INSIG2*	insulin induced gene 2, transcript variant X2	-9.14	1.31E-16	1.78E-12
lcl|AKCR02000014.1_mrna_6101	6	*PRPF40A*	PRP40 pre-mRNA processing factor 40 homolog A, transcript variant X3	-3.86	1.63E-04	3.89E-02
lcl|AKCR02000016.1_mrna_6314	7	*ANXA7*	annexin A7, transcript variant X2	-8.36	2.64E-10	5.99E-07
lcl|AKCR02000016.1_mrna_6316	7	*PPP3CB*	protein phosphatase 3, catalytic subunit, beta isozyme, transcript variant X2	-6.39	1.12E-04	2.87E-02
lcl|AKCR02000017.1_mrna_6671	7	*FGFR2*	fibroblast growth factor receptor 2, transcript variant X6	-5.69	1.13E-04	2.87E-02
lcl|AKCR02000017.1_mrna_6678	7	*ATE1*	arginyltransferase 1, transcript variant X2	-3.96	1.13E-06	8.57E-04
lcl|AKCR02000018.1_mrna_6866	8	*RWDD3*	RWD domain containing 3, transcript variant X1	-3.71	1.58E-04	3.83E-02
lcl|AKCR02000018.1_mrna_6931	8	*DDAH1*	dimethylarginine dimethylaminohydrolase 1	-2.00	8.78E-05	2.54E-02
lcl|AKCR02000022.1_ncrna_7812	12	LOC102098775	uncharacterized LOC102098775	-7.42	1.72E-07	2.13E-04
lcl|AKCR02000023.1_mrna_7908	15	*CMC4*	C-x(9)-C motif containing 4, transcript variant X2	-6.45	1.69E-07	2.13E-04
lcl|AKCR02000029.1_mrna_8762	Z	*NUDT12*	nudix (nucleoside diphosphate linked moiety X)-type motif 12, transcript variant X2	-7.49	3.49E-08	5.93E-05
**lcl|AKCR02000030.1_mrna_8889**	**20**	***SLC2A11B***	**solute carrier family 2, facilitated glucose transporter member 11-like**	**-1.54**	**3.77E-07**	**3.76E-04**
lcl|AKCR02000031.1_mrna_9103	14	*NDRG4*	NDRG family member 4, transcript variant X4	-10.76	6.25E-16	4.25E-12
lcl|AKCR02000033.1_mrna_9571	19	*USP3*	ubiquitin specific peptidase 3, transcript variant X2	-3.28	5.29E-05	1.68E-02
lcl|AKCR02000043.1_mrna_10560	18	*STRBP*	spermatid perinuclear RNA binding protein, transcript variant X5	-6.36	1.73E-04	4.00E-02
lcl|AKCR02000043.1_mrna_10703	18	*FAM73B*	family with sequence similarity 73, member B, transcript variant X2	-7.14	4.08E-07	3.76E-04
lcl|AKCR02000043.1_mrna_10737	18	*ODF2*	outer dense fiber of sperm tails 2, transcript variant X1	-6.29	2.01E-04	4.34E-02
lcl|AKCR02000044.1_ncrna_10984	21	LOC102092662	uncharacterized LOC102092662, transcript variant X7	-1.90	1.47E-04	3.64E-02
lcl|AKCR02000069.1_mrna_13024	7	*UBE2D1*	ubiquitin-conjugating enzyme E2D 1, transcript variant X5	-3.49	1.63E-05	7.94E-03
lcl|AKCR02000081.1_mrna_13565	17	*SETD5*	SET domain containing 5, transcript variant X1	-5.85	1.36E-05	6.88E-03
lcl|AKCR02000084.1_mrna_13655	16	*NFRKB*	nuclear factor related to kappaB binding protein, transcript variant X2	-3.87	4.93E-05	1.64E-02
lcl|AKCR02000093.1_mrna_14043	17	*NR2C2*	nuclear receptor subfamily 2, group C, member 2, transcript variant X1	-6.15	5.29E-06	3.13E-03
lcl|AKCR02000094.1_mrna_14114	2	*PTHB1-like*	protein PTHB1-like	-6.01	1.76E-04	4.00E-02
lcl|AKCR02000096.1_mrna_14183	4	*FNIP2*	folliculin interacting protein 2, transcript variant X1	-5.83	8.38E-05	2.48E-02

lcl|AKCR02000109.1_mrna_14615	11	*NSG2*	neuron-specific protein family member 2, transcript variant X1	-1.63	1.72E-05	8.10E-03
lcl|AKCR02000137.1_mrna_15413	44	*CLCN3*	chloride channel, voltage-sensitive 3, transcript variant X2	-3.39	1.96E-06	1.33E-03
lcl|AKCR02000205.1_mrna_16786	n/a	*CHTOP*	chromatin target of PRMT1, transcript variant X2	-8.91	1.11E-11	3.01E-08
lcl|AKCR02000382.1_ncrna_18045	n/a	LOC102094279	uncharacterized LOC102094279	-6.63	1.03E-05	5.40E-03
lcl|AKCR02000001.1_mrna_663	2	*PCBP3*	poly(rC)-binding protein 3, transcript variant X5	5.97	9.42E-09	1.83E-05
lcl|AKCR02000002.1_mrna_998	3	*RNASET2*	ribonuclease T2, transcript variant X2	3.95	2.52E-07	2.86E-04
lcl|AKCR02000003.1_mrna_1726	1	*IGSF5*	immunoglobulin superfamily, member 5, transcript variant X2	5.19	7.10E-05	2.15E-02
lcl|AKCR02000010.1_mrna_5105	6	*UBR3*	ubiquitin protein ligase E3 component n-recognin 3 (putative), transcript variant X3	5.10	9.68E-05	2.69E-02
lcl|AKCR02000011.1_mrna_5402	5	*SAA1*	serum amyloid A protein-like	1.23	3.24E-05	1.38E-02
lcl|AKCR02000016.1_mrna_6318	7	*PPP3CB*	protein phosphatase 3, catalytic subunit, beta isozyme, transcript variant X1	4.05	4.14E-07	3.76E-04
lcl|AKCR02000019.1_ncrna_7006	19	LOC102088835	uncharacterized LOC102088835	6.01	2.23E-06	1.44E-03
**lcl|AKCR02000030.1_mrna_8893**	**20**	***CHEK2***	**checkpoint kinase 2, transcript variant X3**	**1.82**	**4.93E-05**	**1.64E-02**
**lcl|AKCR02000030.1_mrna_8949**	**20**	***TRAFD1***	**TRAF-type zinc finger domain containing 1, transcript variant X3**	**6.14**	**4.63E-05**	**1.62E-02**
lcl|AKCR02000033.1_mrna_9582	19	*TPM1*	tropomyosin 1 (alpha), transcript variant X1	1.47	8.51E-06	4.83E-03
lcl|AKCR02000042.1_mrna_10423	13	*ECI1*	enoyl-CoA delta isomerase 1, transcript variant X2	6.54	2.88E-12	9.80E-09
lcl|AKCR02000044.1_ncrna_10985	21	LOC102092662	uncharacterized LOC102092662, transcript variant X2	1.93	3.61E-05	1.40E-02
lcl|AKCR02000054.1_mrna_11884	14	*SLC7A6*	solute carrier family 7 (amino acid transporter light chain, y+L system), member 6, transcript variant X3	5.84	3.54E-05	1.40E-02
lcl|AKCR02000071.1_mrna_13251	11	*MED7*	mediator complex subunit 7, transcript variant X2	1.48	4.21E-08	6.37E-05
lcl|AKCR02000084.1_mrna_13658	16	*PRDM10*	PR domain zinc finger protein 10, transcript variant X3	5.08	4.62E-05	1.62E-02
lcl|AKCR02000104.1_ncrna_14427	11	LOC10209304	uncharacterized LOC102093048, transcript variant X2	5.81	2.86E-05	1.26E-02
lcl|AKCR02000140.1_ncrna_15477	1	LOC106146049	uncharacterized LOC106146049	2.51	4.90E-06	3.03E-03
lcl|AKCR02000172.1_mrna_16226	n/a	*HPCA*	hippocalcin	4.38	9.20E-05	2.61E-02
lcl|AKCR02000199.1_mrna_16675	n/a	*RGCC*	regulator of cell cycle	1.57	1.01E-04	2.75E-02
lcl|AKCR02000201.1_mrna_16731	29	*KRT14*	keratin, type I cytoskeletal 14, transcript variant X2	3.91	2.07E-04	4.40E-02
lcl|AKCR02000205.1_mrna_16792	n/a	*S100A16*	S100 calcium binding protein A16	1.95	1.82E-04	4.00E-02
lcl|AKCR02000968.1_ncrna_18603	n/a	LOC106145929	uncharacterized LOC106145929	1.31	1.06E-04	2.83E-02
lcl|AKCR02001022.1_mrna_18627	n/a	*SLC27A2*	very long-chain acyl-CoA synthetase-like	2.91	3.59E-05	1.40E-02
lcl|AKCR02003528.1_mrna_18908	n/a	*TLR1*	toll-like receptor 1	3.57	1.79E-04	4.00E-02
