# Supplementary material for: Molecular parallelisms between pigmentation in the avian iris and the integument of ectothermic vertebrates
Source: PLoS Genet. 2021 Feb 23;17(2):e1009404. doi: 10.1371/journal.pgen.1009404 (PMC7935293; doi:10.1371/journal.pgen.1009404)
Supplement: S4 Table — TPM values were calculated using the quasi-mapping approach implemented in the software Salmon [46]. (PDF) [file pgen.1009404.s007.pdf]

S4 Table. Individual expression values (TPM, transcripts per million) for a set of genes implicated in pterin metabolism and chromatophore differentiation (see also S3 Table). TPM values were calculated using the quasi-mapping approach implemented in the software *Salmo*n [46].

Gene	Transcript	pigeonWHITE1	pigeonWHITE2	pigeonYELLOW1	pigeonYELLOW2	pigeonYELLOW3	pigeonYELLOW4
GCH1	AKCR02000092.1_mrna_14027	28.45	13.64	19.45	19.12	7.80	13.73
PTS	AKCR02000134.1_mrna_15298	74.18	98.50	89.59	85.29	72.31	83.81
SPR	AKCR02000006.1_mrna_3541	1.65	0.00	9.50	23.29	11.96	1.31
AKR1D1	AKCR02000008.1_mrna_4628	23.15	14.66	20.47	17.85	19.88	13.01
AKR1A1	AKCR02000060.1_mrna_12406	44.77	39.21	90.73	92.91	65.49	48.52
CBR1	AKCR02000003.1_mrna_1754	116.27	97.43	99.66	110.61	106.28	98.24
DHFR	AKCR02000088.1_mrna_13862	20.01	22.93	68.12	43.38	30.94	19.48
QDPR	AKCR02000006.1_mrna_3739	23.40	24.00	34.70	27.53	25.86	25.65
PCBD1	AKCR02000016.1_mrna_6518	7.02	12.59	7.93	12.93	7.00	10.38
PCBD2	AKCR02000025.1_mrna_8312	12.05	6.19	23.19	25.02	16.64	8.66
XDH	AKCR02000039.1_mrna_10202	0.40	0.96	3.90	1.65	0.71	0.63
GCHFR	AKCR02000143.1_mrna_15557	9.65	12.52	4.87	2.81	6.21	3.53
CSF1R	AKCR02000025.1_mrna_8384	23.66	22.51	36.89	53.26	30.10	20.18
PAX3	AKCR02000022.1_mrna_7736	3.53	3.72	9.14	14.66	7.53	4.10
PAX7	AKCR02000044.1_mrna_10959	0.00	0.11	0.71	0.00	0.19	0.34
MITF	AKCR02000026.1_mrna_8479	29.45	24.83	58.40	81.16	47.57	27.71
GJB3	AKCR02000153.1_mrna_15891	0.00	0.86	0.00	0.00	0.00	0.00
GPR143	AKCR02000007.1_mrna_4281	53.43	38.57	100.63	111.65	51.77	43.99
SOX5	AKCR02000132.1_mrna_15260	0.46	0.60	4.33	4.09	1.85	0.84
SOX10	AKCR02000009.1_mrna_4858	24.36	18.22	22.40	38.39	20.31	21.58
TH	AKCR02000011.1_mrna_5378	0.38	0.49	1.62	0.13	0.17	0.46
