# Supplementary material for: Molecular parallelisms between pigmentation in the avian iris and the integument of ectothermic vertebrates
Source: PLoS Genet. 2021 Feb 23;17(2):e1009404. doi: 10.1371/journal.pgen.1009404 (PMC7935293; doi:10.1371/journal.pgen.1009404)
Supplement: Attachment — Submitted filename: andrade_et_al_review.docx [file pgen.1009404.s008.docx]

In this manuscript by Andrade et al, the authors map the Mendelian *pearl eye* locus in domestic pigeons (which causes complete loss of pterin pigmentation in the iris) to a single locus containing the gene *SLC2A11B.* They find that presence of a premature stop codon in this gene correlates perfectly with the trait, and follow up with RNA-sequencing data from the irises of *pearl eye* versus wild-type birds to show that this genetic model is the most likely one.

Artificial selection for various traits in pigeons, many of them Mendelian, has long been a favorite topic for biologists. I enjoyed reading this work and I believe others will too. In addition to linking phenotype to genotype, it also raises interesting questions about how cell types such as xanthophores and irridophores have evolved (the authors close by noting that the work on such cell types in fish could inform future work in birds, but I suspect that the converse will also be true, and that many interesting differences will be found that lead to a better general understanding).

I would be happy to consider a revised version of this manuscript that addresses the following two main points:

**Main points:**

1) The data in Figures 2-3 make a solid case that there is a single causative locus, which contains *SLC2A11B*, and the data in Figure 4 A-C make a plausible case that the G/A point mutation in *SLC2A11B* is the causative variant.

However, in my opinion the authors take the wrong approach with Figure 4 D-E in that the alternative hypotheses they are emphasizing are too specific (i.e. they restrict those panels to genes that are known from previous work to be involved in pterin synthesis or pigment cell specification). Certainly those are good guesses, and important to consider. But what if pterin pigmentation genetics in the pigeon iris works a little differently than in other model systems? Better to consider a generic cis-acting regulatory variant that resides within that locus.

There are probably many approaches to disproving this hypothesis of a regulatory variant, but the following seems to me the most straightforward: First the authors should take Figure 4A and restrict it to genes that are on the same chromosome as the GWAS peak (or use two colors to denote genes on the same vs different chromosome). This should be possible by using the Holt et al genetic map (https://doi.org/10.1534/g3.117.300443). That analysis should become a new panel in Figure 4. My guess is that there won't be many genes other than *SLC2A11B* on that chromosome that are significantly down-regulated in *tr* irises. Secondly, the authors should take the hits from Supplementary Table 3 and determine which chromosome each one is on. Any hit that is on a different chromosome than the *tr* locus can be safely ruled out as a causative gene under a cis-regulatory model. This will make the case for *SLC2A11B* much stronger.

2) The title needs to change. The current one would be a nice title for a "News and Views" piece covering this paper, but I feel it is not appropriate for the paper itself, since there is hardly any new data presented from ectothermic vertebrates (although the new data presented shed interesting light on previous work on these species).

**Minor points:**

1) Figure 2: It looks like there is a region on the 11th scaffold from the left (in yellow) that shows some association signal, although it does not reach Bonferroni level (panel A) as well as reduced nucleotide diversity (panel C). Do you know what is in there, and is it worth commenting on?
2) Figure 3A: Why does the panel indicate that the causative haplotype shared by the *tr/tr* birds contains only reference alleles? I see in the Shapiro et al genome paper that the bird they used was a Danish Tumbler, which from what I've been able to gather is homozygous for *tr* (https://www.angelfire.com/ga/huntleyloft/eye.html). I would assume that's the reason. Perhaps that's worth mentioning at some point in the text, because it's a little confusing at first glance.
3) Figure 3A: This panel needs a horizontal scale in kb, and gene models plotted to scale.
4) Figure 3B: This panel needs a gene model, preferably with codons denoted, so it is more apparent that the candidate causative mutation causes a premature stop codon.
5) Figure 4C: At this point in the paper it is probably premature to label the reads as *Tr* and *tr.* They should be labeled according to nucleotide or amino acid encoded.
6) Figure 4C: For each bird a single datum is presented (a proportion of reads) so there is no need to denote it with a yellow bar next to a red bar; just use one bar.
7) Supplementary Figure 2: Perhaps a Sashimi Plot based on reads mapped to the reference genome would be a better way of conveying the gene structure and splice variants.
8) It seems there are some additional xanthophore/pigment cell markers such as *aox5, gjp8*, and *mitfa* that you might wish to include in your analysis from the recent Lencer et al preprint: https://doi.org/10.1101/2020.12.24.424338.
9) Lines 230-232: Novelty claims are difficult to verify and should generally be avoided.
