# Supplementary material for: Molecular parallelisms between pigmentation in the avian iris and the integument of ectothermic vertebrates
Source: PLoS Genet. 2021 Feb 23;17(2):e1009404. doi: 10.1371/journal.pgen.1009404 (PMC7935293; doi:10.1371/journal.pgen.1009404)
Supplement: Attachment — Submitted filename: Andrade_et_al_eyecolor_response.doc [file pgen.1009404.s009.doc]

Dear Dr. Kelly A. Dyer and Dr. Bret Payseur,

Following the helpful comments from three reviewers on our manuscript “**Molecular parallelisms between pigmentation in the avian iris and the integument of ectothermic vertebrates**” (manuscript ID: PGENETICS-D-20-01786) we produced a revised version with the appropriate corrections and clarifications. All suggestions from the reviewers were taken into consideration, below you can find comments on each remark. We hope that with these changes you find our manuscript to be ready for publication in *PLoS Genetics*.

**Reviewer #1**

*“The only small reservation I had concerned the presentation of candidate pteridine and chromatophore associated gene expression in Figure 4D,E, in which there were some trends but not significant differences. Since these data are not normalized by cell type, but instead represent a complex mixture of cells in the iris, and sample sizes are very small, they really do not contribute substantially. So I would be more inclined to move these panels to a supplementary figure, with the appropriate caveats so they are not over-interpreted by readers.”*

This is a good point. We agree with Reviewer #1 that the interpretation of these findings should take into account the reduced sample size and the fact that the RNAseq analysis was done at the bulk tissue level. We have now added a brief cautionary note to the “Results” section explicitly mentioning these caveats, in order to alert the reader to these issues (page 10, lines 225-227). We also moved panels D and E from Figure 4 to Supporting information (now Supplementary Figure 3).

**Reviewer #2**

*“My only suggestion would be to add somewhere (likely figure 3?) the intron-exon structure of the SLC2A11B (and any other nearby genes). Basically the results refer to SLC2A11B as being the only gene in this region, however the reader never really gets to 'see' this result directly, as well as any other nearby genes. Thus, including this annotation information visually would be an improvement.”*

Following the suggestion of Reviewer #2 (see also comment #5 from Reviewer #3), we have revised Figure 3 (adding a new panel B) to illustrate the location of genes in the interval in which we performed our identical-by-descent analysis. This should help the reader to visualize that *SLC2A11B* is the only gene contained within the target interval, to the exclusion of other genes in the vicinity.

**Reviewer #3**

*1. “However, in my opinion the authors take the wrong approach with Figure 4 D-E in that the alternative hypotheses they are emphasizing are too specific (i.e. they restrict those panels to genes that are known from previous work to be involved in pterin synthesis or pigment cell specification). Certainly those are good guesses, and important to consider. But what if pterin pigmentation genetics in the pigeon iris works a little differently than in other model systems? Better to consider a generic cis-acting regulatory variant that resides within that locus.*

*There are probably many approaches to disproving this hypothesis of a regulatory variant, but the following seems to me the most straightforward: First the authors should take Figure 4A and restrict it to genes that are on the same chromosome as the GWAS peak (or use two colors to denote genes on the same vs different chromosome). This should be possible by using the Holt et al genetic map (https://doi.org/10.1534/g3.117.300443). That analysis should become a new panel in Figure 4. My guess is that there won't be many genes other than SLC2A11B on that chromosome that are significantly down-regulated in tr irises. Secondly, the authors should take the hits from Supplementary Table 3 and determine which chromosome each one is on. Any hit that is on a different chromosome than the tr locus can be safely ruled out as a causative gene under a cis-regulatory model. This will make the case for SLC2A11B much stronger.”*

The reviewer suggests the possibility of a *cis*-acting regulatory variant in a gene other than *SLC2A11B* causing the pearl-eye phenotype. Overall, we feel that our claim that the identified nonsense mutation (AKCR02000030.1:1,895,934) is likely the causal mutation for this phenotype is strong, for the following reasons: 1) an 8.5 kb interval containing only *SLC2A11B* is the only genomic segment in which all pearl-eye pigeons are homozygous, while wild-type pigeons are either heterozygous or homozygous alternative; 2) within this interval, this is the only point mutation with a major effect on protein function; 3) this position is highly conserved across birds; 4) assembled transcripts and gene expression patterns support a scenario of nonsense mediated mRNA decay in pearl-eye pigeons.

However, we do agree that other non-identified regulatory mutations (affecting either this gene or others near this locus) could potentially also contribute to the phenotype. Following the reviewer’s suggestion, we have inspected our differential expression results to identify genes that localize to the same genomic scaffold as *SLC2A11B*. These are now highlighted in Supplementary Table 3. Out of a total of 64 differentially expressed genes, only two apart from *SLC2A11B* belonged to scaffold 30: *TRAFD1* (located ca. 1.6 Mb from the candidate interval) and *CHEK2* (located ca. 4 kb away). Both genes were upregulated in pearl-eye iris, in contrast to *SLC2A11B*. While *CHEK2* is located in the vicinity of our candidate causal 8.5 kb haplotype, its known functions are associated with response to DNA damage, so with current information it is difficult to make a point that this gene could be linked to differences in pigmentation. This is in direct contrast with *SLC2A11B*, which has been implicated in dermal pigmentation in fish. These results are now explicitly mentioned in the “Results” section (pages 8-9, lines 192-198). It now reads: “Two other genes located in the same scaffold as *SLC2A11B* were also differentially expressed: *TRAFD1* (located ca. 1.6 Mb from the candidate interval) and *CHEK2* (located ca. 4 kb away). While we cannot rule out the possibility of *cis*-regulatory variants within our candidate region affecting the expression of these nearby genes (particularly for *CHEK2*), their known functions do not suggest any direct link to pigment synthesis, transport or deposition that could explain differences in eye color.”

*2. “The title needs to change. The current one would be a nice title for a "News and Views" piece covering this paper, but I feel it is not appropriate for the paper itself, since there is hardly any new data presented from ectothermic vertebrates (although the new data presented shed interesting light on previous work on these species)”*

While we understand the point made by the reviewer, we feel that our current title describes well our original hypothesis that led us to initiate this work, which was that of investigating the genetics of avian eye color to understand if and how the avian iris and the integument of ectothermic vertebrates are linked. This was a relationship that had already been implied in the literature, and molecular signatures of this link are confirmed through our results. For these reasons, we feel that our proposed title has a direct connection to the hypothesis being tested, so we have chosen to keep the original formulation. However, if the editors and the reviewer feel strongly on this point, we will not object changing it, and would welcome suggestions in this regard.

*3. "Figure 2: It looks like there is a region on the 11th scaffold from the left (in yellow) that shows some association signal, although it does not reach Bonferroni level (panel A) as well as reduced nucleotide diversity (panel C). Do you know what is in there, and is it worth commenting on?”*

The reviewer raises a valid point. We have revisited the results of our analyses to confirm whether this second genomic signal on scaffold 11 (and another on scaffold 7) holds a signal of genetic association to the phenotype. We retrieved genotypes for the SNPs with the highest significance scores in our association analysis in both regions, and confirmed if genotypes of pearl-eye and wild-type pigeons in our dataset behave according to the expectations of a single locus with an autosomal recessive inheritance pattern. We found a moderate difference in allele frequencies; however, this is more likely explained by genetic structure in our sample, rather than any true relationship to the phenotype, since the individual genotypes did not follow the expected inheritance pattern. We have added a brief sentence describing these results (pages 6-7, lines 142-144). It reads: “Two additional regions, on scaffolds 7 and 11, evidenced a moderate (non-significant) signal of association in our genomic scans, but individual genotypes in both regions did not follow the expected inheritance pattern”.

*4. “Figure 3A: Why does the panel indicate that the causative haplotype shared by the tr/tr birds contains only reference alleles? I see in the Shapiro et al genome paper that the bird they used was a Danish Tumbler, which from what I've been able to gather is homozygous for tr (https://www.angelfire.com/ga/huntleyloft/eye.html). I would assume that's the reason. Perhaps that's worth mentioning at some point in the text, because it's a little confusing at first glance.”*

As the reviewer correctly points out, the reference genome for the rock pigeon was assembled from an individual with the pearl-eye phenotype. This means that the wild-type allele is considered to be the alternative (non-reference) allele in Figure 3A. Although we mention this in the main body of text (in particular, in the “Methods” section), we agree that this situation may cause confusion to some readers, so we clarified this in the caption to this figure.

*5. “Figure 3A: This panel needs a horizontal scale in kb, and gene models plotted to scale.”*

As stated above (see response to Reviewer #2), we changed Figure 3 by adding a new panel (B in the revised version) to include the location of the genes in the interval of our identical-by-descent analysis.

*6. “Figure 3B: This panel needs a gene model, preferably with codons denoted, so it is more apparent that the candidate causative mutation causes a premature stop codon.”*

We agree with this suggestion. We have revised Figure 3 (panel C in the revised version) to include a model showing the 5’ end of exon 3 of *SLC2A11B* and additional details on the corresponding amino acid content. This now highlights the location of the premature STOP codon in the mutant mRNA.

*7. “Figure 4C: At this point in the paper it is probably premature to label the reads as Tr and tr. They should be labeled according to nucleotide or amino acid encoded.”*

We agree with the reviewer and changed the legend to Figure 4C accordingly.

*8. “Figure 4C: For each bird a single datum is presented (a proportion of reads) so there is no need to denote it with a yellow bar next to a red bar; just use one bar.”*

We agree with the reviewer and modified Figure 4C accordingly by changing the graphical representation to a stacked bar chart, which hopefully should convey more correctly the proportion of reads of each allele at the candidate mutation in the three heterozygous birds.

*9. “Supplementary Figure 2: Perhaps a Sashimi Plot based on reads mapped to the reference genome would be a better way of conveying the gene structure and splice variants.”*

Following this suggestion by Reviewer #3, we edited Supplementary Figure 2 to include an additional panel (B), with a Sashimi plot for each of the two birds that were used for transcriptome assembly (one wild-type and one pearl-eye, both homozygous). We maintained the original sequence alignment as panel (A), since we believe it is also informative about the exon structure of the wild-type and pearl-eye transcripts when compared to the better annotated genomes of the chicken and zebra finch.

*10. “It seems there are some additional xanthophore/pigment cell markers such as aox5, gjp8, and mitfa that you might wish to include in your analysis from the recent Lencer et al preprint: https://doi.org/10.1101/2020.12.24.424338.”*

In accordance to the suggestion made by the reviewer, we have now included extra data in this figure, which has been moved to supporting information (Supplementary Figure 3), as suggested by Reviewer #1. From the genes suggested by the reviewer, we added data for *MITF* and *GJB3* (avian orthologue of the zebrafish *gjb8*), while *AOX5* (xanthine dehydrogenase) was already present in our figure (as *XDH*, following an alternate nomenclature). We also added data for *GPR143*, an additional gene mentioned in the Lencer et al. preprint, and added this reference to the main text.

*11. “Lines 230-232: Novelty claims are difficult to verify and should generally be avoided.”*

This is a good point and we have now modified the sentence to highlight the contribution of our manuscript to coloration research in vertebrates (page 10, lines 238-240). It now reads: “These results are confirmed in an independent study by [26], and contribute towards an increased understanding of the biology of pigmentation in vertebrates.”
