# Supplementary material for: Molecular parallelisms between pigmentation in the avian iris and the integument of ectothermic vertebrates
Source: PLoS Genet. 2021 Feb 23;17(2):e1009404. doi: 10.1371/journal.pgen.1009404 (PMC7935293; doi:10.1371/journal.pgen.1009404)
Supplement: Attachment — Submitted filename: Andrade_et_al_eyecolor_response2.doc [file pgen.1009404.s010.doc]

Dear Dr. Kelly A. Dyer and Dr. Bret Payseur,

Following a second round of helpful comments from one of the three reviewers on our manuscript “**Molecular parallelisms between pigmentation in the avian iris and the integument of ectothermic vertebrates**” (manuscript ID: PGENETICS-D-20-01786R2) we produced a revised version with the appropriate corrections and clarifications. This second round of comments followed from a previous suggestion that was not correctly attended to on our part, so we hope that now we have managed to successfully answer the reviewer’s criticisms. Details on the answer can be seen below.

**Reviewer #3**

*“Reviewer #3: The authors have responded to main point #1 by highlighting the differential expression hits in Supplementary Table 3 that are on the same scaffold as SLC2A11B. This is a step in the right direction. However, this is not what I had originally suggested: I had suggested the authors determine which genetic linkage group each hit belongs to, not which scaffold (indeed the latter is already clear from the table). This is because linkage groups typically span a whole chromosome whereas scaffolds do not. Granted, the scaffold the causative haplotype resides on is a large one (8.6 Mb), and the haplotype is about 1.8 Mb from the scaffold end (so it does seem likely that any putative cis-regulated gene would be located on the scaffold). However, given that A) enhancers can sometimes act at megabase distances, and B) scaffolds are sometimes misassembled, it does not seem unreasonable to be curious whether any of the differential expression hits reside on different scaffolds, which are linked to the one in question. To illustrate what I mean, I have attached the results of a simple analysis, where I downloaded Table S6 from the Holt et al paper, which contains a list of scaffolds and their corresponding linkage groups, and did a simple data join in R to determine the linkage group for each of the genes in Supplementary Table 3. SLC2A11B is on linkage group 20 and indeed the only other hits on that linkage group are on the same scaffold (CHEK2 and TRAFD1). This corresponds to what the authors noted in their response to Main Point #1 (but note the improvement in that we can now make a statement about the entire linkage group). Of the 64 genes, the linkage group for all but 8 could be determined from the Holt et al results. This seems to me to be worth including in the paper. Given that it is such an easy thing to include, I cannot recommend publication until the authors either include it or explain why it should not be included.*

*Aside from that, I have no further issues to bring up. The matter of the title I will leave to the authors and editors. This was a fun paper to read. I think if the linkage group data is included it will be even better.”*

We thank the reviewer for the extra care taken with this issue. We apologize that we did not interpret the initial suggestion correctly by only taking into the scaffold to where the candidate causal locus belongs. As suggested by the reviewer, Supplementary Table 3 has now been updated, including a new column with information on the linkage group of each differentially expressed gene (following the genetic map of Holt et al., 2018). Confirming the analysis run by the reviewer, we found that only 3 genes out of the 64 were located in the linkage group where the candidate causal locus resides. In addition, and in parallel to the analysis of the reviewer, we found that 8 of the genes were not assigned to any linkage group. To complement the changes made to this supplementary table, we also changed the corresponding paragraph on the main text (Page 8, Line 194). We hope that these changes answer the remaining criticism satisfactorily, but we are fully available to revisit these analysis if needed.
